# The Mg member of the isotypic series *M*Te_6_O_13_

**DOI:** 10.1107/S1600536813003875

**Published:** 2013-02-13

**Authors:** Mahdi Shirkhanlou, Matthias Weil

**Affiliations:** a Institute for Chemical Technologies and Analytics, Division of Structural Chemistry, Vienna University of Technology, Getreidemarkt 9/164-SC, A-1060 Vienna, Austria

## Abstract

MgTe_6_O_13_, magnesium hexa­tellurate(IV), is isotypic with the structures of divalent first-row transition metal analogues *M*Te_6_O_13_ (*M* = Mn, Fe, Co, Ni and Zn). The asymmetric unit contains one Mg, two Te and five O atoms of which the Mg and one O atom lie on a threefold rotation axis. The structure is made up from slightly distorted [MgO_6_] octa­hedra (isolated from each other), distorted [TeO_4_] bis­phenoids and [TeO_4 + 1_] tetra­gonal pyramids sharing corners and edges. This arrangement leads to the formation of a dense three-dimensional structure.

## Related literature

The title compound is isotypic with its Co, Mn, Ni (Irvine *et al.*, 2003 ▶), Fe (van der Lee & Astier, 2007 ▶; van der Lee, 2013 ▶) and Zn (Nawash *et al.*, 2007 ▶) analogues. For other phases in the system Mg–Te–O, see: Trömel & Ziethen-Reichnach (1970 ▶). For structure determinations of MgTe_2_O_5_ and Mg_2_Te_3_O_8_, see: Weil (2005 ▶) and Lin *et al.* (2013 ▶), respectively. The crystal chemistry of oxotellurates(IV) has been reviewed by Zemann (1971 ▶).

## Experimental

### 

#### Crystal data

MgTe_6_O_13_
*M* *_r_* = 997.91
Trigonal, 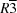

*a* = 10.1676 (2) Å
*c* = 18.9701 (3) Å
*V* = 1698.39 (5) Å^3^
*Z* = 6
Mo *K*α radiation
μ = 15.38 mm^−1^
*T* = 295 K
0.22 × 0.14 × 0.09 mm

#### Data collection

Bruker APEXII CCD diffractometer
Absorption correction: multi-scan (*SADABS*; Bruker, 2012 ▶) *T*_min_ = 0.437, *T*_max_ = 0.749
50742 measured reflections
2875 independent reflections
2764 reflections with *I* > 2σ(*I*)
*R*_int_ = 0.044

#### Refinement

*R*[*F* ^2^ > 2σ(*F* ^2^)] = 0.017
*wR*(*F* ^2^) = 0.034
*S* = 1.36
2875 reflections
62 parameters
Δρ_max_ = 1.17 e Å^−3^
Δρ_min_ = −1.40 e Å^−3^

### 

Data collection: *APEX2* (Bruker, 2012 ▶); cell refinement: *SAINT* (Bruker, 2012 ▶); data reduction: *SAINT*; program(s) used to solve structure: *SHELXS97* (Sheldrick, 2008 ▶); program(s) used to refine structure: *SHELXL97* (Sheldrick, 2008 ▶); molecular graphics: *ATOMS for Windows* (Dowty, 2008 ▶); software used to prepare material for publication: *publCIF* (Westrip, 2010 ▶).

## Supplementary Material

Crystal structure: contains datablock(s) I, global. DOI: 10.1107/S1600536813003875/pj2001sup1.cif

Structure factors: contains datablock(s) I. DOI: 10.1107/S1600536813003875/pj2001Isup2.hkl

Additional supplementary materials:  crystallographic information; 3D view; checkCIF report

## Figures and Tables

**Table 1:** Structural data of isotypic *M*Te_6_O_13_ compounds (Å, Å^3^) in space group *R* 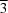

*M* ^2+^	*a*	*c*	*V*
Mg*^*a*^*	10.1676 (2)	18.9701 (3)	1698.39 (5)
Mn*^*b*^*	10.2505 (5)	19.2195 (9)	1748.89 (14)
Fe*^*c*^*	10.16630 (10)	18.9330 (3)	1694.63 (4)
Co*^*b*^*	10.1641 (5)	18.9814 (9)	1698.23 (14)
Ni*^*b*^*	10.1522 (5)	18.8669 (9)	1684.30 (14)
Zn*^*d*^*	10.1283 (9)	18.948 (3)	1683.3 (3)

Notes: (*a*) this work; (*b*) Irvine *et al.* (2003 ▶); (*c*) van der Lee & Astier (2007 ▶); (*d*) Nawash *et al.* (2007 ▶).

**Table 2:** Selected bond lengths (Å)

Mg1—O5	2.0676 (12)
Mg1—O2^i^	2.1596 (12)
Te1—O3	1.8524 (12)
Te1—O2	1.9324 (11)
Te1—O1	2.1314 (2)
Te1—O2^ii^	2.1842 (11)
Te2—O5	1.8489 (11)
Te2—O4	1.9186 (11)
Te2—O4^iii^	2.0286 (12)
Te2—O3	2.2118 (12)
Te2—O5^iv^	2.5908 (12)

Symmetry codes: (i) 
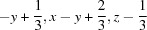
; (ii) 
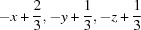
; (iii) 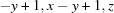; (iv) 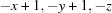.

**Fig. 1.**
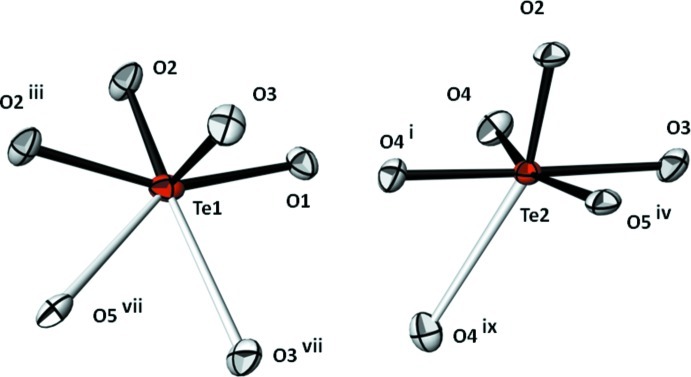
The coordination spheres of the two tellurium(IV) atoms. Te—O bonds < 2.6 Å are given as solid lines, Te—O bonds between 2.6 and 3.1 Å as open lines; probability level of the displacement ellipsoids is 74%. [Symmetry codes: i) -y + 1, x-y+1, z; iii) -x + 2/3, -y + 1/3, -z + 1/3; iv) -x + 1, -y + 1, -z; vii) y, -x+y, -z; ix) x-y+2/3, x + 1/3, -z + 1/3.]

**Fig. 2.**
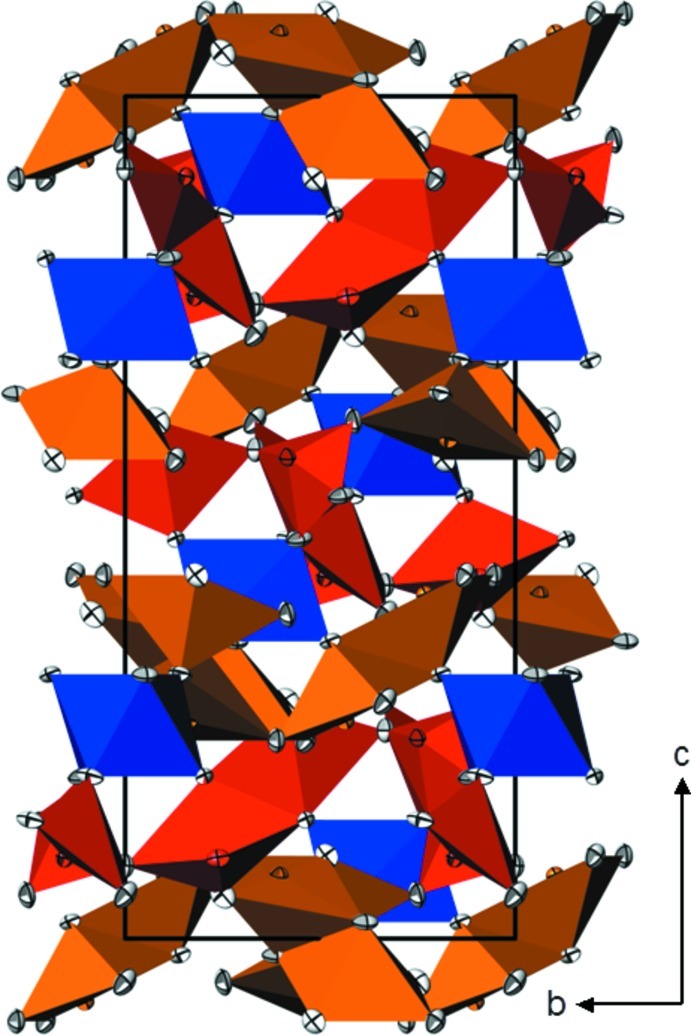
The crystal packing of the MgTe6O13 structure in a view along [100]. [MgO6] polyhedra are blue, [Te1O4] polyhedra are red and [Te2O4 + 1] polyhedra are orange. Probability level as in Fig. 1.

## supplementary crystallographic information

### Comment

Single crystals of Mg_2_Te_3_O_8_ (Lin *et al.*, 2013) and of the title compound, MgTe_6_O_13_, were obtained during hydrothermal phase formation studies in the system Mg–Se–Te–O. These two phases, along with MgTe_2_O_5_ have already been described as existing phases in the system Mg–Te–O and were characterized by their X-ray diffraction patterns at that time (Trömel & Ziethen-Reichnach, 1970). Whereas for MgTe_2_O_5_ (Weil, 2005) and Mg_2_Te_3_O_8_ (Lin *et al.*, 2013) structure determinations from single-crystal data were reported, detailed structure data for MgTe_6_O_13_ were missing up to now.

MgTe_6_O_13_ is a member of the isotypic series *M*Te_6_O_13_ crystallizing in space group *R*3. Structure determinations were reported for the Co, Mn, Ni (Irvine *et al.*, 2003), Fe (van der Lee & Astier, 2007) and Zn (Nawash *et al.*, 2007) members. It should be mentioned that the given space group *R*3*m* for FeTe_6_O_13_ (van der Lee & Astier, 2007) is incorrect. The correct space group in fact is *R*3 (van der Lee, 2013). Lattice parameters and volumes of the several *M*Te_6_O_13_ phases are in a narrow range (Table 1), as one expects from the similar ionic radii of Mg and the first row transition metals.

The magnesium cation is located on a threefold rotation axis and is surrounded by six oxygen atoms in a slightly distored octahedral environment. Because of the high tellurium content in the structure, the [MgO_6_] octahedra are isolated from each other. The two distinct tellurium(IV) atoms exhibit different oxygen environments. Te1 is bonded to four oxygen atoms in form of a bisphenoid with distances ranging from 1.8524 (12) to 2.1842 (11) Å. Considering Te···O separations less than 3.1 Å, two remote O atoms (O3, O5) are also present, leading to a pentagonal pyramid [TeO_4 + 2_] as the resulting coordination polyhedron. Atom Te2 is bonded to four O atoms with distances ranging from 1.8489 (11) to 2.2118 (12) Å, with an additional O atom 2.5908 (12) Å away. The resulting [TeO_4 + 1_] polyhedron is a distorted tetragonal pyramid. It is augmented to a distorted octahedron if the remote O4 atom at a distance of 3.0387 (12) Å is also considered. For both [TeO*_x_*] polyhedra (Fig. 1, Table 2) the rules derived by Zemann for the crystal chemistry of oxotellurates(IV) are valid (Zemann, 1971).

The [MgO_6_], [Te1O_4_] and [Te2O_4 + 1_] building units share corners and edges, thereby forming a dense three-dimensional structure (Fig. 2). In a simpler view, the structure can be described as being built up from distorted hexagonal layers of the Mg and Te atoms extending parallel to (001) and stacked in an *ABCA'B'C'* sequence along [001]. The oxygen atoms are situated in the voids of this arrangement.

### Experimental

Magnesium oxide, tellurium dioxide and selenic acid (conc.; 96%_wt_) were loaded in the stoichiometric ratio 3:2:1 in a Teflon lined stainless steel autoclave (overall volume 10 ml) that was filled up to two-thirds of its volume with water. The autoclave was then heated at 503 K for one week. Few colourless single crystals of Mg_2_Te_3_O_8_ (Lin *et al.*, 2013) and MgTe_6_O_13_, both with a platy habit, were isolated from the colourless solid reaction product. X-ray powder diffraction (XRPD) of the ground bulk material revealed Mg_2_Te_3_O_8_ and TeO_2_ as main products. Se-containing phases could not be identified by XRPD in the solid reaction product.

### Refinement

The atomic coordinates of isotypic ZnTe_6_O_13_ (Nawash *et al.*, 2007) were used as starting parameters for the refinement. Reflection 003 was affected from the beamstop and was omitted from the refinement. The highest positive and negative residual electron densities are located 0.68 and 0.48 Å, respectively, from atom Te1.

### Crystal data

**Table:**

MgTe_6_O_13_	*D*_x_ = 5.854 Mg m^−^^3^
*M**_r_* = 997.91	Mo *K*α radiation, λ = 0.71073 Å
Trigonal, *R*3	Cell parameters from 9908 reflections
Hall symbol: -R 3	θ = 3.2–43.5°
*a* = 10.1676 (2) Å	µ = 15.38 mm^−^^1^
*c* = 18.9701 (3) Å	*T* = 295 K
*V* = 1698.39 (5) Å^3^	Plate, colourless
*Z* = 6	0.22 × 0.14 × 0.09 mm
*F*(000) = 2568	

### Data collection

**Table:**

Bruker APEXII CCD diffractometer	2875 independent reflections
Radiation source: fine-focus sealed tube	2764 reflections with *I* > 2σ(*I*)
Graphite monochromator	*R*_int_ = 0.044
ω– and φ–scans	θ_max_ = 43.6°, θ_min_ = 2.6°
Absorption correction: multi-scan (*SADABS*; Bruker, 2012)	*h* = −19→19
*T*_min_ = 0.437, *T*_max_ = 0.749	*k* = −19→19
50742 measured reflections	*l* = −36→36

### Refinement

**Table:**

Refinement on *F*^2^	Primary atom site location: isomorphous structure methods
Least-squares matrix: full	*w* = 1/[σ^2^(*F*_o_^2^) + 7.6037*P*] where *P* = (*F*_o_^2^ + 2*F*_c_^2^)/3
*R*[*F*^2^ > 2σ(*F*^2^)] = 0.017	(Δ/σ)_max_ = 0.002
*wR*(*F*^2^) = 0.034	Δρ_max_ = 1.17 e Å^−^^3^
*S* = 1.36	Δρ_min_ = −1.40 e Å^−^^3^
2875 reflections	Extinction correction: *SHELXL97* (Sheldrick, 2008), Fc^*^=kFc[1+0.001xFc^2^λ^3^/sin(2θ)]^-1/4^
62 parameters	Extinction coefficient: 0.000970 (18)
0 restraints	

### Special details

**Table:**

Geometry. All e.s.d.'s (except the e.s.d. in the dihedral angle between two l.s. planes) are estimated using the full covariance matrix. The cell e.s.d.'s are taken into account individually in the estimation of e.s.d.'s in distances, angles and torsion angles; correlations between e.s.d.'s in cell parameters are only used when they are defined by crystal symmetry. An approximate (isotropic) treatment of cell e.s.d.'s is used for estimating e.s.d.'s involving l.s. planes.
Refinement. Refinement of *F*^2^ against ALL reflections. The weighted *R*-factor *wR* and goodness of fit *S* are based on *F*^2^, conventional *R*-factors *R* are based on *F*, with *F* set to zero for negative *F*^2^. The threshold expression of *F*^2^ > σ(*F*^2^) is used only for calculating *R*-factors(gt) *etc*. and is not relevant to the choice of reflections for refinement. *R*-factors based on *F*^2^ are statistically about twice as large as those based on *F*, and *R*- factors based on ALL data will be even larger.

### Fractional atomic coordinates and isotropic or equivalent isotropic displacement parameters (Å^2^)

**Table:**

	*x*	*y*	*z*	*U*_iso_*/*U*_eq_	
Mg1	0.3333	0.6667	−0.07728 (5)	0.00655 (14)	
Te1	0.237607 (11)	0.082435 (11)	0.095241 (5)	0.00766 (2)	
Te2	0.394752 (11)	0.496265 (11)	0.075896 (5)	0.00633 (2)	
O1	0.0000	0.0000	0.08633 (11)	0.0085 (3)	
O2	0.20309 (13)	0.11297 (14)	0.19252 (6)	0.01008 (18)	
O3	0.25075 (15)	0.25178 (13)	0.05181 (7)	0.01187 (19)	
O4	0.20451 (14)	0.48320 (14)	0.09819 (6)	0.00998 (17)	
O5	0.38463 (14)	0.52546 (14)	−0.01971 (6)	0.00969 (17)	

### Atomic displacement parameters (Å^2^)

**Table:**

	*U*^11^	*U*^22^	*U*^33^	*U*^12^	*U*^13^	*U*^23^
Mg1	0.0066 (2)	0.0066 (2)	0.0065 (3)	0.00329 (11)	0.000	0.000
Te1	0.01113 (4)	0.00635 (4)	0.00675 (4)	0.00530 (3)	0.00084 (2)	0.00065 (2)
Te2	0.00718 (4)	0.00727 (4)	0.00516 (3)	0.00408 (3)	−0.00023 (2)	0.00038 (2)
O1	0.0068 (4)	0.0068 (4)	0.0118 (7)	0.0034 (2)	0.000	0.000
O2	0.0065 (4)	0.0145 (5)	0.0060 (4)	0.0028 (4)	0.0005 (3)	−0.0017 (3)
O3	0.0137 (5)	0.0063 (4)	0.0146 (5)	0.0042 (4)	−0.0030 (4)	0.0017 (3)
O4	0.0090 (4)	0.0112 (4)	0.0123 (4)	0.0069 (4)	0.0017 (3)	0.0017 (3)
O5	0.0143 (5)	0.0116 (4)	0.0055 (4)	0.0083 (4)	−0.0002 (3)	0.0014 (3)

### Geometric parameters (Å, º)

**Table:**

Mg1—O5^i^	2.0676 (12)	Te1—O2^vi^	2.1842 (11)
Mg1—O5^ii^	2.0676 (12)	Te1—O3^vii^	2.9337 (13)
Mg1—O5	2.0676 (12)	Te1—O5^vii^	3.0387 (12)
Mg1—O2^iii^	2.1596 (12)	Te2—O5	1.8489 (11)
Mg1—O2^iv^	2.1596 (12)	Te2—O4	1.9186 (11)
Mg1—O2^v^	2.1596 (12)	Te2—O4^i^	2.0286 (12)
Te1—O3	1.8524 (12)	Te2—O3	2.2118 (12)
Te1—O2	1.9324 (11)	Te2—O5^viii^	2.5908 (12)
Te1—O1	2.1314 (2)	Te2—O4^ix^	3.0560 (12)
			
O5^i^—Mg1—O5^ii^	94.67 (5)	Te1^x^—O1—Te1^xi^	119.377 (15)
O5^i^—Mg1—O5	94.67 (5)	Te1—O2—Mg1^xii^	130.51 (6)
O5^ii^—Mg1—O5	94.67 (5)	Te1—O2—Te1^vi^	105.30 (5)
O5^i^—Mg1—O2^iii^	99.09 (5)	Mg1^xii^—O2—Te1^vi^	122.67 (6)
O5^ii^—Mg1—O2^iii^	76.38 (5)	Te1—O2—Te2^vi^	94.79 (5)
O5—Mg1—O2^iii^	164.10 (5)	Mg1^xii^—O2—Te2^vi^	84.08 (4)
O5^i^—Mg1—O2^iv^	76.38 (5)	Te1^vi^—O2—Te2^vi^	78.72 (4)
O5^ii^—Mg1—O2^iv^	164.10 (5)	Te1—O2—Te1^xi^	75.79 (4)
O5—Mg1—O2^iv^	99.09 (5)	Mg1^xii^—O2—Te1^xi^	74.86 (4)
O2^iii^—Mg1—O2^iv^	91.88 (5)	Te1^vi^—O2—Te1^xi^	140.31 (5)
O5^i^—Mg1—O2^v^	164.10 (5)	Te2^vi^—O2—Te1^xi^	140.94 (4)
O5^ii^—Mg1—O2^v^	99.09 (5)	Te1—O2—Te2^xiii^	124.64 (5)
O5—Mg1—O2^v^	76.38 (5)	Mg1^xii^—O2—Te2^xiii^	77.64 (3)
O2^iii^—Mg1—O2^v^	91.88 (5)	Te1^vi^—O2—Te2^xiii^	81.17 (4)
O2^iv^—Mg1—O2^v^	91.88 (5)	Te2^vi^—O2—Te2^xiii^	139.31 (3)
O3—Te1—O2	102.12 (6)	Te1^xi^—O2—Te2^xiii^	67.54 (2)
O3—Te1—O1	82.59 (5)	Te1—O3—Te2	130.72 (6)
O2—Te1—O1	82.98 (6)	Te1—O3—Te1^xiv^	120.60 (6)
O3—Te1—O2^vi^	90.88 (5)	Te2—O3—Te1^xiv^	105.58 (4)
O2—Te1—O2^vi^	74.70 (5)	Te1—O3—Te1^xi^	91.57 (5)
O1—Te1—O2^vi^	154.89 (5)	Te2—O3—Te1^xi^	107.62 (5)
O3—Te1—O3^vii^	81.28 (3)	Te1^xiv^—O3—Te1^xi^	87.42 (3)
O2—Te1—O3^vii^	172.70 (4)	Te2—O4—Te2^ii^	136.27 (7)
O1—Te1—O3^vii^	91.12 (6)	Te2—O4—Te2^xiii^	106.42 (5)
O2^vi^—Te1—O3^vii^	111.91 (4)	Te2^ii^—O4—Te2^xiii^	103.43 (4)
O3—Te1—O5^vii^	89.65 (5)	Te2—O4—Te1^xi^	115.96 (5)
O2—Te1—O5^vii^	130.80 (4)	Te2^ii^—O4—Te1^xi^	98.97 (4)
O1—Te1—O5^vii^	146.21 (6)	Te2^xiii^—O4—Te1^xi^	82.34 (3)
O2^vi^—Te1—O5^vii^	57.32 (4)	Te2—O4—Te2^i^	71.27 (4)
O3^vii^—Te1—O5^vii^	55.14 (3)	Te2^ii^—O4—Te2^i^	70.62 (3)
O5—Te2—O4	95.29 (5)	Te2^xiii^—O4—Te2^i^	104.98 (3)
O5—Te2—O4^i^	94.31 (5)	Te1^xi^—O4—Te2^i^	168.27 (4)
O4—Te2—O4^i^	93.39 (7)	Te2—O5—Mg1	133.10 (7)
O5—Te2—O3	85.36 (5)	Te2—O5—Te2^viii^	105.65 (5)
O4—Te2—O3	83.67 (5)	Mg1—O5—Te2^viii^	111.82 (5)
O4^i^—Te2—O3	177.00 (5)	Te2—O5—Te1^xiv^	112.67 (5)
O5—Te2—O5^viii^	74.35 (5)	Mg1—O5—Te1^xiv^	94.65 (4)
O4—Te2—O5^viii^	166.09 (5)	Te2^viii^—O5—Te1^xiv^	90.25 (3)
O4^i^—Te2—O5^viii^	96.57 (5)	Te2—O5—Te2^i^	71.20 (4)
O3—Te2—O5^viii^	86.22 (5)	Mg1—O5—Te2^i^	68.01 (4)
O5—Te2—O4^ix^	160.15 (4)	Te2^viii^—O5—Te2^i^	114.99 (4)
O4—Te2—O4^ix^	72.54 (5)	Te1^xiv^—O5—Te2^i^	153.01 (4)
O4^i^—Te2—O4^ix^	71.41 (4)	Te2—O5—Te2^ii^	71.12 (4)
O3—Te2—O4^ix^	108.16 (4)	Mg1—O5—Te2^ii^	67.95 (4)
O5^viii^—Te2—O4^ix^	119.92 (3)	Te2^viii^—O5—Te2^ii^	172.20 (5)
Te1—O1—Te1^x^	119.377 (15)	Te1^xiv^—O5—Te2^ii^	97.55 (3)
Te1—O1—Te1^xi^	119.377 (15)	Te2^i^—O5—Te2^ii^	57.381 (17)

Symmetry codes: (i) −*y*+1, *x*−*y*+1, *z*; (ii) −*x*+*y*, −*x*+1, *z*; (iii) −*y*+1/3, *x*−*y*+2/3, *z*−1/3; (iv) *x*+1/3, *y*+2/3, *z*−1/3; (v) −*x*+*y*+1/3, −*x*+2/3, *z*−1/3; (vi) −*x*+2/3, −*y*+1/3, −*z*+1/3; (vii) *y*, −*x*+*y*, −*z*; (viii) −*x*+1, −*y*+1, −*z*; (ix) *x*−*y*+2/3, *x*+1/3, −*z*+1/3; (x) −*x*+*y*, −*x*, *z*; (xi) −*y*, *x*−*y*, *z*; (xii) *x*−1/3, *y*−2/3, *z*+1/3; (xiii) *y*−1/3, −*x*+*y*+1/3, −*z*+1/3; (xiv) *x*−*y*, *x*, −*z*.

### Structural data of isotypic *M*Te_6_O_13_ compounds (Å, Å^3^) in space group *R*3

**Table:**

*M*^2+^	*a*	*c*	*V*
Mg*^a^*	10.1676 (2)	18.9701 (3)	1698.39 (5)
Mn*^b^*	10.2505 (5)	19.2195 (9)	1748.89 (14)
Fe*^c^*	10.16630 (10)	18.9330 (3)	1694.63 (4)
Co*^b^*	10.1641 (5)	18.9814 (9)	1698.23 (14)
Ni*^b^*	10.1522 (5)	18.8669 (9)	1684.30 (14)
Zn*^d^*	10.1283 (9)	18.948 (3)	1683.3 (3)

Notes: (*a*) this work; (*b*) Irvine *et al.* (2003); (*c*) van der Lee & Astier (2007); (*d*) Nawash *et al.* (2007).
